# *Staphylococcus aureus* Infections in the Paediatric Intensive Care Unit: Illustrated Cases

**DOI:** 10.1155/2021/6661932

**Published:** 2021-06-04

**Authors:** Kam Lun Hon, Ronald C. M. Fung, Karen K. Y. Leung, Alexander K. C. Leung, Wun Fung Hui, Wing Lum Cheung

**Affiliations:** ^1^Paediatric Intensive Care Unit, Department of Paediatrics and Adolescent Medicine, Hong Kong Children's Hospital, Hong Kong, Hong Kong; ^2^Department of Pediatrics, The University of Calgary and The Alberta Children's Hospital, Calgary, Alberta T2M 0H5, Canada

## Abstract

*Staphylococcus aureus* is known to be one of the most common gram-positive microorganisms and an important pathogen associated with sepsis and toxic shock. We present four anonymized consecutive cases in a paediatric intensive care unit (PICU) to illustrate the different clinical manifestations of staphylococcal infections, including local infection versus systemic infection, toxic shock versus septic shock, and osteomyelitis. Eczema, short gut syndrome, and scald injury may be associated. Haematologic and coagulopathic abnormalities may be present. Prompt diagnosis and use of appropriate antimicrobial treatments is essential to reducing mortality and morbidity associated with staphylococcal infections.

## 1. Introduction

*Staphylococcus aureus* is known to be one of the most common gram-positive microorganisms and an important pathogen associated with sepsis and toxic shock in paediatric patients requiring intensive care [1]. There are different strains of *Staphylococcus aureus* which can cause a wide range of infections due to its extensive virulence factors [2]. We present four consecutive cases to illustrate the different clinical manifestations of staphylococcal infections, including local infection versus systemic infection, toxic shock versus septic shock, and osteomyelitis [2]. Prompt diagnosis and use of appropriate antimicrobial treatments is essential to reducing mortality and morbidity associated with staphylococcus infections.

## 2. Case Series

### 2.1. Case 1: An Infant with Eczema, Methicillin-Sensitive *Staphylococcus aureus*, and Transient Erythroblastopenia of Childhood

A 6-month-old boy with eczema presented with high fever (40°C), tachycardia (heart rate 200 per minute), oliguria, and respiratory failure. He was treated with cefotaxime for presumed septic shock. Echocardiography showed mildly impaired left ventricular function. He was also noted to have hemoglobin 7.7 g/dL, absolute neutrophil 0.37 × 10^9^/L, and platelet 117,000/mm^3^. He was suspicious of bone marrow failure, possibly consistent with transient erythroblastopenia of childhood (TEC) or hemophagocytic lymphohistiocytosis (HLH) or malignancy since he had gross hepatosplenomegaly and high ferritin (1283 pmol/L), though normal fibrinogen and triglyceride. An urgent bone marrow examination revealed pure red-cell aplasia with granulocytic maturation arrest and reactive changes but no malignant infiltration. The patient was stabilized with mechanical ventilation, red-cell transfusion, antibiotics, and low-dose adrenaline infusion. He was also noted to have an ulcerated lesion over his left forearm. The family had two cats, and they lived in a village house. Bed bugs were also found to be present in old furniture. Investigations did not show evidence of scrub typhus, dengue fever, or bartonella; but throat swab and left forearm wound swab revealed heavy growth of methicillin-sensitive *Staphylococcus aureus* (MSSA) and scant growth of *Staphylococcus epidermidis*; the minimum inhibitory concentrations are 0.25 *μ*g/ml and ≤0.12 *μ*g/ml, respectively. The child's condition gradually improved after 4 days of treatment. He was able to be extubated and weaned off inotropic support. Haemoglobin, neutrophil, and platelet counts normalized over a week.

### 2.2. Case 2: An Infant with Methicillin-Sensitive *Staphylococcus aureus* Osteomyelitis

**Figure 1:**
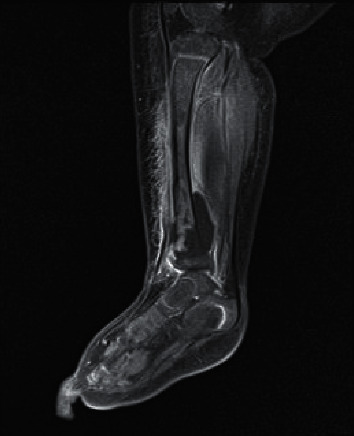
Osteomyelitis changes in the right tibial region by MRI.

A 6-month-old infant girl, with a history of eczema and short-gut syndrome due to neonatal volvulus, requiring parenteral nutrition, was admitted to a PICU for septic shock and right ankle cellulitis. She had fever (temperature of 38.1°C), tachycardia (heart beat 190 per minute), and a red swollen right ankle. Blood culture from her central venous catheter yielded methicillin-sensitive *Staphylococcus aureus* (MSSA). Magnetic resonance imaging (MRI) of her right ankle showed right distal tibial osteomyelitis, extending into the epiphyseal plate with a large subperiosteal collection around the distal tibia (Figure 1). The patient was treated with vancomycin and clindamycin. Urgent right tibial debridement was performed. A 6-week course of cloxacillin was added.

Pus was drained from the subperisosteal space, and cultures grew MSSA. She remained hemodynamically stable, with normal hematologic, renal, and liver functions. After the antibiotic therapy and the debridement, she returned to baseline functioning and was discharged from the PICU 3 days later to the surgical team to complete a 6-week course of antibiotics.

### 2.3. Case 3: Scald Injury Complicated by Staphylococcal Toxic Shock Syndrome

**Figure 2:**
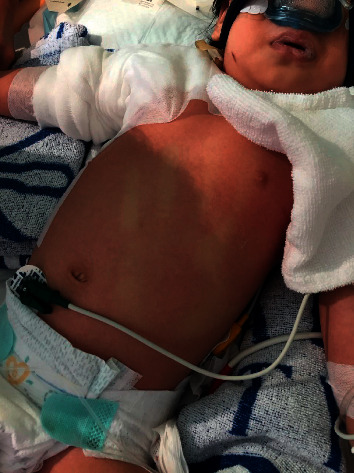
Deteriorated central capillary refill with palm mark on the torso.

A previously healthy 21-month-old girl presented with fever and shock shortly after wound debridement for her scald burn, which was sustained to the right neck, right shoulder, and scapular region down to the right arm. The burn, sustained after accidentally pouring a boiling congee over herself, was of partial thickness (second degree) and covered 5% body surface area. She was initially admitted to the surgical ward, where she had the blisters deroofed and the wounds dressed in sterile saline gauzes. Wound debridement was scheduled to be performed in the operation theatre 3 days later. However, she developed a fever of up to 38.8°C just before the debridement and the temperature remained elevated (38.7°C) the night after the operation. She had marked tachycardia (150-190/min) and hypotension (89/40 mm Hg), and capillary refill was 5 seconds. She was lethargic and required 1 L/min nasal oxygen to maintain the saturation. She was noted to have diffuse erythroderma over her trunk. She was suspected to have toxic shock syndrome and was transferred to the PICU for immediate intervention, including fluid resuscitation and broad-spectrum antibiotics, cefotaxime, and vancomycin. She required inotropic support with dopamine, adrenaline, and noradrenaline. The vasoactive inotropic score was up to 35. Echocardiography revealed mildly impaired left ventricular function. She was intubated for suspected acute respiratory distress syndrome (lowest PaO_2_/FiO_2_ 61.5, FiO_2_ 1.0). Intravenous immunoglobulin (1 g/kg/day) was given for two consecutive days. Multisystem involvement was evident from elevated serum creatinine kinase 5019 IU/L, raised serum alanine aminotransferase 65 IU/L, lowplatelet count 89,000/mm^3^ (hematologic), and alterations in consciousness (central nervous system). Capillary refill time over the right sole and left chest rash was 10 seconds and 2 seconds, respectively. The capillary refill became 5 seconds over the chest whilst the rash evolved (Figure 2). Wound swab culture grew MSSA. Blood cultures remained negative. This girl met criteria for staphylococcal toxic shock syndrome, as per the 2011 case definition from the Centers for Disease Control and Prevention (CDC). Other blood investigations showed serum lactate 3.8 mmol/L, serum procalcitonin 103.07 ng/ml, C-reactive protein 246 mg/L, prolonged prothrombin time 25.3 seconds, and activated partial thromboplastin time 53 seconds with normal fibrinogen. The girl's condition gradually stabilized with mechanical ventilation, inotropic support, and antibiotic therapy. She was eventually extubated and weaned off all inotropes after 4 days.

### 2.4. Case 4: A teenager with Toxic Shock and Methicillin-Resistant *Staphylococcus aureus* Bacteriaemia

A previously healthy 12-year-old girl presented with a 3-day history of fever, menorrhagia, and a painful left submandibular swelling. She had a mixed picture of hemorrhagic shock and septic shock, as evidenced by tachycardia, hypotension, and anaemia (hemoglobin 4.1 g/dL). Respiratory, hepatic, and neurological functions were not affected. She immediately received fluid resuscitation, red-cell transfusion, noradrenaline infusion, and broad-spectrum antibiotic therapy including cefotaxime and vancomycin. Apart from anaemia, blood investigations revealed neutropenia (zero absolute neutrophil), lymphopenia (absolute lymphocyte 0.1 × 10^9^/L), and thrombocytopenia (platelet 19,000/mm^3^). Serum amylase was normal (61 IU/L). The blood culture was positive for methicillin-resistant *Staphylococcus aureus* (MRSA, Panton–Valentine leucocidin negative). Serologic evaluation ruled out Epstein–Barr virus, cytomegalovirus, measles, varicella-zoster virus, parvovirus, and human immunodeficiency virus. Computed tomography of the neck and thorax showed diffuse left parotid swelling with reactive inflammatory features and enlarged left upper cervical lymph nodes without abscess. Bone marrow examination revealed a markedly hypocellular marrow without hemopoietic elements, blasts, or abnormal infiltrates. The girl's condition stabilized, and she was discharged from the PICU after 4 days.

## 3. Discussion

*Staphylococcus aureus* is the leading cause of infection in critical-care settings accounting for significant morbidity and mortality [3]. It is capable of causing a wide range of infections in humans, from life-threatening infections in otherwise healthy individuals to nosocomial infections complicating the clinical course of patients with other primary medical or surgical disease processes [4, 5]. There are many different strains of *Staphylococcus aureus*, and there is controversy over whether all strains are equally pathogenic or whether the invasive disease is associated with particular virulent genotypes [5]. The five stages in the pathogenesis of *Staphylococcus aureus* infections are colonization, local infection, systemic disseminated infection and/or sepsis, metastatic infection, and toxinosis. Some of these stages can be clearly identified and delineated in the cases we reviewed (Table 1) [2].

### 3.1. Local Infection with Systemic Effects

In the case of the infant with eczema (Case 1), the patient's presentation was atypical as he presented with septic shock, pancytopenia, and gross hepatosplenomegaly; hence, malignancy was suspected and supportive treatment for sepsis was started. Although only the wound swab was positive for *Staphylococcus aureus*, the septic shock was likely a result of the systemic effects from the exfoliative toxins. Transient erythroblastopenia of childhood (TEC) is an uncommon benign normocytic anaemia. Although it was observed that TEC often follows a viral-like illness, no specific aetiologic agent was identified [6]. We were unable to find any literature reporting the association of TEC with *Staphylococcus aureus* infections. Furthermore, TEC is uncommon in infants less than 1 year of age, and most patients with TEC are hemodynamically stable [6]. Therefore, we are of the view that TEC could be a coincidental finding in our patient with eczema infected by *Staphylococcus aureus*. *Staphylococcus aureus* is an important infectious pathogen in children with eczema, and the clinical features (especially severity and lesion intensity) are useful indicators in “predicting” moderate-to-heavy *Staphylococcus aureus* colonization and infection in children with eczema [7–9].

### 3.2. Systemic Disseminated Infection and Osteomyelitis

In Case 2, osteomyelitis was immediately suspected as the infant presented with septic shock and right ankle cellulitis. Osteomyelitis was the result of haematogenous dissemination of staphylococcal infection [10]. Early diagnosis is the cornerstone of the successful management of this disease [10]. In view of the history of eczema and need for long-term central-line use, longer duration of cloxacillin coverage is required. Empiric antibiotic treatment consists of a short intravenous cycle based on antistaphylococcal penicillin or a cephalosporin in children aged over 3 months, with the addition of gentamicin in infants aged under 3 months. An oral regimen may also be an option depending on the bioavailability of the antibiotic chosen and the assessment of clinical and laboratory data [10].

### 3.3. Local Infection and Toxic Shock Syndrome

For Case 3, the predisposing factor for toxic shock syndrome (TSS) was the scald injury. TSS is primarily the result of a superantigen-mediated cytokine storm and *M*-protein-mediated neutrophil activation, resulting in the release of mediators leading to respiratory failure, vascular leakage, and shock [11]. It is caused by the toxin-producing strains of *Staphylococcus aureus* and *Streptococcus pyogenes* [12]. Management of TSS should be aggressive. The majority of patients with TSS will require admission to an intensive care unit for supportive care, particularly in the case of multiple organ failure [12–14]. A surveillance study in the UK reported that about 8% of TSS were associated with burns; hence, physicians should be on alert that this can be a possible cause in children with burns who suddenly become unwell [12, 14]. Antibiotic treatment should cover both *Staphylococcus aureus* and *Streptococcus pyogenes*, for example, a combination of cephalosporin, penicillin, and vancomycin. The addition of clindamycin or gentamicin can also reduce toxin production and mortality [15, 16]. Furthermore, it is also important to remove the foci of bacterial toxin production as the outcomes are worse in patients who do not have the source of infection removed [12, 13]. Although the use of intravenous immune globulin (IVIG) therapy in children with TSS has been controversial, the use of IVIG may be considered in patients with severe staphylococcal TSS who are unresponsive to other therapeutic measures even though the optimal IVIG regimen has not been sufficiently investigated [17].

### 3.4. Systemic Infection with MRSA

In Case 4, the teenager had hemorrhagic and septic shock due to MRSA bacteraemia. Pancytopenia led to a blood marrow examination that revealed aplastic anaemia. Apart from MRSA, other infectious causes for aplastic anaemia were not found. Infection is a major cause of death for patients with aplastic anaemia, while infections associated with neutropenia are a major cause of morbidity and mortality in this patient population [18]. Recovery from neutropenia is directly related to survival, and therefore, aplastic anaemia patients who are severely neutropenic should ideally be nursed in isolation, while prophylactic antibiotics and antifungals should also be considered [19, 20].

## 4. Conclusions

*Staphylococcus aureus* is an important human pathogen, and paediatricians must not overlook its potential to inflict significant morbidity and mortality [21]. The four cases illustrate severe staphylococcal disease affecting children of all ages by different pathogenesis and clinical manifestations, including local infection versus systemic infection, toxic shock versus septic shock, and osteomyelitis (Table 1). Eczema, short-gut syndrome, and scald injury may be associated. Haematologic and coagulopathic abnormalities may be present. Prompt diagnosis with aggressive support treatments and antimicrobials are essential in managing and improving clinical outcomes of these critically ill children.

## Figures and Tables

**Table 1:** *Staphylococcus aureus* disease in the PICU.

	Case 1	Case 2	Case 3	Case 4
Gender	Male	Female	Female	Female

Age	5 months	6 months	21 months	12 years

Premorbid	Eczema	Short-gut syndrome, Broviac line, and eczema	Scald injury	Healthy

Organ system				

Cardiovascular	Impaired LV function	Normal	Impaired LV function. Low diastolic pressure	Low diastolic pressure

Pulmonary	Mechanical ventilation for respiratory failure	Normal	Intubated for ARDS	Normal

Haematologic	Pancytopenia with gross hepatosplenomegaly. Normal clotting	Normal	Thrombocytopenia. Prolonged PT and aPTT	Pancytopenia. Aplastic anaemia. Normal clotting

Renal	Normal	Normal	Normal	Normal

Hepatic	Normal	Normal	Raised ALT	Normal

CNS	Normal	Normal	Lethargic	Normal

Sites of *Staphylococcus aureus*	Throat swab (MSSA) + wound swab (*S. epidermidis*)	Blood culture from Broviac line (MSSA), subperiosteal abscess (MSSA)	Wound swab (MSSA)	Blood culture (MRSA)

Final diagnosis	Septic shock and TEC, possibly associated with bed bug bite and MSSA	MSSA, osteomyelitis	MSSA, toxic shock syndrome following scald	MRSA septicemia associated with aplastic anaemia

ARDS: acute respiratory distress syndrome. LV: left ventricular. MSSA: methicillin-sensitive *Staphylococcus aureus*. MRSA: methicillin-resistant *Staphylococcus aureus*. TEC: transient erythroblastopenia of childhood.

## Data Availability

Vital signs and some blood parameters were included in this retrospective review of anonymized cases.
